# Exploring the Psychometric Properties of a Cumulative Adversity Assessment in a Diverse Sample of Adults

**DOI:** 10.1093/geroni/igaf122.2705

**Published:** 2025-12-31

**Authors:** Samantha Corley, Frank Mann, Stacey Scott

**Affiliations:** Stony Brook University, Stony Brook, New York, United States; Renaissance School of Medicine at Stony Brook University, Stony Brook, New York, United States; Stony Brook University, Stony Brook, New York, United States

## Abstract

Cumulative adversity, defined as the accumulation of adverse life events across a lifetime, has been associated with worse mental and physical health. Black and Hispanic Americans exhibit increased risk for exposure to adverse experiences, however, these groups have been underrepresented in the development of cumulative adversity assessments. This study aimed to explore the prevalence of adverse life events and the psychometric properties of a cumulative adversity assessment, originally posited by Turner, Wheaton, and Lloyd (1995), in a racially and ethnically diverse sample (N = 260). Factor analysis models were estimated and compared, uncovering a two-factor solution, encompassing unintentional and intentional trauma. Prevalence rates of cumulative adversity were relatively low in the sample, with specific events surrounding parents and spouses being the most common. Significant associations between demographic characteristics and these factors were identified through Weighted Least Squares (WLS) regression analyses, shedding light on race/ethnicity and gender disparities for adversity, as well as relationships with age, household composition, work, and marital status. Findings contribute to a deeper understanding of cumulative lifetime adversity, providing insights by increasing the applicability of the lifetime adversity measurement in diverse populations in future studies.

